# Comparison of clinical outcomes after endoscopic submucosal dissection and surgery in the treatment of early gastric cancer: A single-institute study

**DOI:** 10.1097/MD.0000000000007210

**Published:** 2017-07-28

**Authors:** Ji Young Chang, Ki-Nam Shim, Chung Hyun Tae, Ko Eun Lee, Jihyun Lee, Kang Hoon Lee, Chang Mo Moon, Seong-Eun Kim, Hye-Kyung Jung, Sung-Ae Jung, Joo-Ho Lee, Min-Sun Cho

**Affiliations:** a Department of Internal Medicine; b Surgery; c Pathology, Ewha Womans University College of Medicine, Seoul, Korea.

**Keywords:** early gastric cancer, endoscopic submucosal dissection, gastrectomy, treatment outcome

## Abstract

The feasibility of expanding the indications for endoscopic submucosal dissection to treat early gastric cancer based on long-term outcomes has shown conflicting results. This study aimed to investigate whether outcomes or adverse events associated with endoscopic submucosal dissection are comparable to those of surgery for early gastric cancer that including the absolute and expanded indications.

Data of 159 early gastric cancers from 153 patients treated with endoscopic submucosal dissection or surgery between January 2004 and October 2014 were reviewed retrospectively. Early gastric cancers fulfilled the absolute or expanded indications with differentiated type adenocarcinoma were included.

The endoscopic submucosal dissection and surgery group showed no significant difference in the incidence of residual disease (*P* = .48), local recurrence (*P* = .46), and metachronous cancer (*P* = .22). Kaplan–Meier analysis showed no significant difference in 2-year (97.6% versus [vs] 92.4%; *P* = .45) and 5-year (95.8% vs 95.6%; *P* = .26) overall survival rate between 2 groups. There was also no significant difference in 2-year (100% vs 94.1%; *P* = .98) and 5-year (100% vs 98.4%; *P* = .89) disease-free survival rate. Early and late adverse events also showed no significant differences.

For the treatment of early gastric cancer fulfilled absolute and expanded indications, endoscopic submucosal dissection is not inferior modality regarding the clinical outcomes and safety, compared with surgery.

## 1 Introduction

Based on Korean cancer statistics for 2012, gastric cancer is the most common cancer among men, and the fourth most common cancer among women.^[1]^ However, the incidence of gastric cancer has been decreasing,^[2]^ whereas the incidence of early gastric cancer (EGC) has increased by up to 50% since nationwide gastric cancer screening was started in 1999.^[3–5]^ The conventional standard treatment for gastric cancer is surgery. With the advancement of instruments and endoscopic techniques, endoscopic submucosal dissection (ESD) was developed for the purpose of en bloc resection, and it is accepted as treatment of choice for EGC without the risk of lymph node metastasis.^[6–8]^ However, because the absolute indications suggested in the Japanese gastric cancer treatment guidelines are designed for endoscopic mucosal resection (EMR), they may be too strict^[9]^ when ESD is a widely available option. The expanded indications for ESD proposed by Gotoda et al^[10]^ and Soetikno et al^[11]^ are based on the very low risk of lymph node metastasis demonstrated in the pathologic results of EGC patients who had undergone gastrectomy with lymph node dissection, including (1) mucosal cancer without ulcer finding irrespective of tumor size, (2) mucosal cancer with an ulcer ≤3 cm in diameter, and (3) minute (<500 μm from the muscularis mucosa) submucosal invasive cancer ≤3 cm in diameter and without an ulcer. Several studies have reported that the short-term clinical outcomes of ESD for EGC that meet expanded indications are comparable to those of absolute indications considering en bloc resection and complete resection.^[6,12]^ A few studies also have discussed the feasibility of expanding indications for ESD to treat EGC based on long-term outcomes,^[12,13]^ whereas others have shown conflicting results suggesting that lymph node metastasis may occur even in cases meeting the expanded indications.^[14,15]^

Therefore, additional clinical studies, including pathological studies, are needed before the expanded indications can be used as the standard treatment guideline. The major concerns of expanding indications are the risk of lymph node metastasis, long-term follow-up outcomes, and adverse events related to the procedure. Thus, we aimed to investigate whether long-term outcomes and adverse events of ESD are comparable to those of surgery for EGC including the absolute and expanded indications.

## 2 Materials and methods

### 2.1 Study population

**Figure 1:**
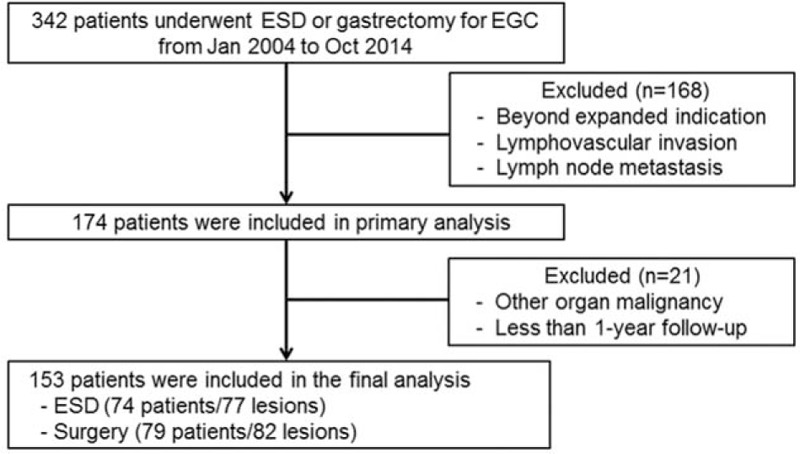
Flowchart of patients included in this study. EGC = early gastric cancer, ESD = endoscopic submucosal dissection.

We retrospectively reviewed the medical records of 153 patients who underwent ESD or gastrectomy with lymph node dissection for EGC at Ewha Womans University Hospital between January 2004 and October 2014, because ESD had been wildly available only since 2004. Patients who met the following inclusion criteria were included in the study: (1) older than 20 years, (2) newly diagnosed with EGC without previous treatment, (3) mucosal cancer without ulcer, irrespective of the tumor size, (4) mucosal cancer with ulcer, ≤ 3 cm, (5) minute (submucosal 1 [SM 1], <500 μm from the muscularis mucosa) submucosal invasive cancer, ≤3 cm, and (6) histologically differentiated-type adenocarcinoma. We excluded patients with lymphovascular invasion or lymph node metastasis and those who were beyond the expanded indications for ESD. Patients who had a less than 1-year duration of follow-up were also excluded. This is illustrated in a fully detailed flowchart shown in Fig. 1. All patients enrolled in the study underwent abdominal-pelvic computed tomography (CT) before treatment for staging. This study was approved by the Institutional Review Board of our medical center (IRB number; 2015-05-020-001).

### 2.2 Treatment methods

All ESDs were performed by 3 experienced endoscopists with a standard single-channel endoscope. First, several marking dots were circumferentially made outside the target lesion by using argon plasma coagulation. Then, a saline solution mixed with epinephrine (0.01 mg/mL) and 0.8% indigo carmine was injected into the submucosal layer to lift the lesion from the muscle layer. Direct submucosal dissection was performed using many types of knives. Finally, endoscopic hemostasis was performed with hemostatic forceps or hemoclips in cases with active bleeding or those with exposed vessels.

Radical gastrectomy with lymph node dissection was performed by 2 experienced surgeons. The extent of lymph node dissection was more than D1+beta (resection of perigastric node stations with left gastric [station 7], common hepatic [station 8a], and celiac arteries [station 9] lymph nodes), and reconstruction methods were decided according to the extent of surgery; Billroth I or II for subtotal gastrectomy, and Roux-en-Y esophagojejunostomy for total gastrectomy.

### 2.3 Histological evaluation

A single pathologist who specialized in gastric cancer retrospectively reviewed all specimens from enrolled patients for lateral and vertical margins of the excision and depth of tumor invasion. SM1 gastric cancer is defined as carcinoma invading less than 500 μm from the muscularis mucosa according to the Japanese Gastric Cancer Handling Codes.^[16]^ Complete resection is defined as (1) >2 mm disease-free lateral margin and >0.5 mm vertical margin in cases where en bloc resection was performed, and (2) complete resection of the entire lesion with sufficient disease-free margins after reconstruction of the entire tissue if tumors were resected in a piecemeal pattern. Ulcers were diagnosed pathologically and in cases in which a definite mucosal defect was detected on endoscopy. Histological subgroups were classified on the basis of the World Health Organization classification of gastric cancer.^[17]^ Well- or moderately differentiated tubular adenocarcinoma and papillary adenocarcinoma were included as the differentiated-type. Poorly differentiated adenocarcinoma, signet ring cell carcinoma, and mucinous adenocarcinoma were defined as the undifferentiated-type.^[18]^

### 2.4 Evaluation of outcomes

The presence of a residual tumor, tumor recurrence, and mortality were analyzed retrospectively. Tumors detected at the resection site within 12 months of the treatment were defined as residual tumors, despite the pathological results showing a complete resection margin at the conclusion of the primary treatment. Local recurrence was diagnosed histologically as the presence of a recurrent tumor at the resection site after 12 months of the treatment. New gastric cancers detected at any other site of the primary resection margin in the remnant stomach were defined as metachronous tumors. Mortality was defined as death from any cause; however, specific disease-related mortality was defined as death due to recurrent or metachronous cancers. Overall survival (OS) was defined as survival from the time of treatment to death due to any cause, whereas disease-specific survival was defined as survival from the time of treatment to gastric cancer-related death. Disease-free survival (DFS) was determined from the time of treatment to cancer recurrence, occurrence of a new gastric cancer, or death from any cause.

Complications were analyzed considering the onset time and on the basis of the Clavien–Dindo classification of surgical complications.^[19]^ An early adverse event was defined as a complication that occurred within 3 months of treatment, whereas late complications were defined as occurring beyond 3 months of treatment. Clinically significant bleeding was defined as bleeding with symptoms such as shock or melena and requiring transfusion or either endoscopic or surgical intervention. Complications were classified into the following 5 grades according to the Clavien–Dindo classification. Grade I complications include any deviation from the normal course that does not require pharmacological treatment or surgical, endoscopic or radiologic intervention. These complications require therapies including only antiemetics, antipyretics, analgesics, and diuretics. Grade II complications include those requiring, pharmacological treatment with drugs other than those allowed for grade I complications as well as blood transfusion or parenteral nutrition. Grade III a complications require a surgical, endoscopic, or radiologic intervention that does not require general anesthesia. Grade III b requires a surgical, endoscopic, or radiologic intervention that requires general anesthesia. Grade IV complications are life-threatening complications involving single organ dysfunction (IV a) or multiorgan dysfunction (IV b). Death is classified as the Grade V complication.

### 2.5 Follow-up after treatment

Follow-up data including recurrence and mortality were collected until October 2015. Routine endoscopy was recommended at 3, 6, and 12-month post-treatment, and annually thereafter for the ESD group. Endoscopy was recommended at 6 and 12-month after surgery and annually thereafter. Abdominal-pelvic CT was recommended every 6 months for 1 year and annually thereafter.

### 2.6 Statistical analysis

All statistical analyses were performed with using SPSS program, version 22.0. Continuous variables were reported as the median with the interquartile range (IQR). To analyze the baseline, clinicopathological characteristics, and adverse events between the 2 groups, the *t-*test or the Mann-Whitney *U* test, were used for continuous variables and the chi-square or the Fisher's exact test for categorical variables. Survival curves were constructed by using the Kaplan–Meier method and compared with the log-rank test. A *P* value of <.05 was considered significant.

## 3 Results

### 3.1 Baseline and clinicopathological characteristics

A total of 159 EGCs in 153 patients were included in this study. The baseline and clinicopathological characteristics of the study population are summarized in Table 1. In total, 74 patients underwent ESD (the ESD group) and 79 patients underwent surgery (the surgery group). The median follow-up duration was 2 years (IQR: 1–5) in the ESD group and 3 years (IQR: 2–6) in the surgery group (*P* = .01). In the surgery group, 74 (93.7%) patients underwent subtotal gastrectomy, and 5 (6.3%) underwent total gastrectomy, with laparoscopic surgery being performed in 67 (84.8%) patients and open surgery in 12 (15.2%) patients. Tumors in the ESD group were significantly smaller compared to those in the surgery group (9 mm vs 15 mm, respectively; *P* < .001), and most of them were located in the lower third of the stomach (*P* < .001). The lesions that fulfilled expanded indications were more prevalent in the surgery group; however, the difference was not significant. Pretreatment histologic results revealed a high number of adenocarcinomas in the surgery group (*P* < .001). There were no significant differences in other characteristics including age, sex, comorbidities, tumor differentiation, macroscopic morphology, invasion depth, or presence of ulceration between the 2 groups.

**Table 1:**
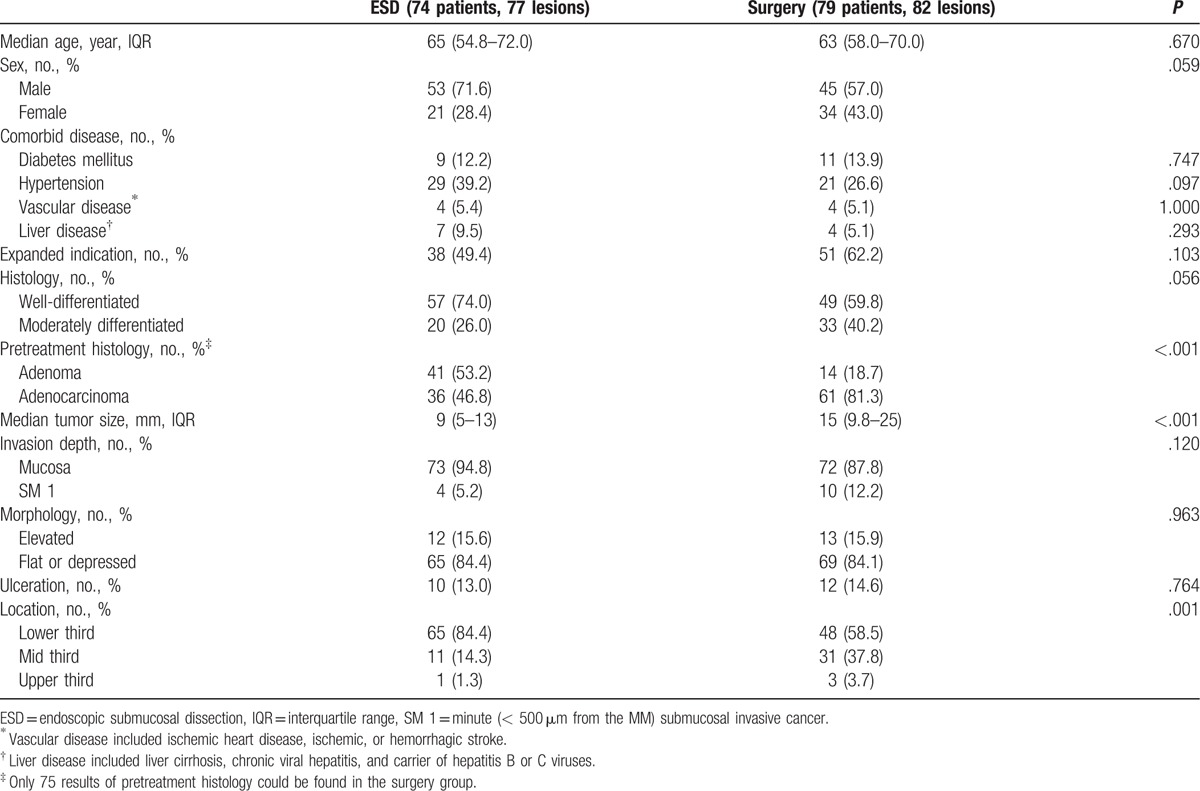
Baseline and clinicopathological characteristics.

### 3.2 Short-term outcomes of ESD

For 77 specimens from 74 patients in the ESD group, the en bloc resection rate was 92.2%, and the complete resection rate was 93.5%. There was 1 case of residual disease in a patient who underwent ESD; no patient in the surgery group was found to have residual disease (*P* = .48). Although complete resection was confirmed at the time of ESD, a 65-year-old man was diagnosed with a residual lesion 6 months after treatment. The primary lesion was an approximately 1.5 cm, flat, elevated, EGC type IIa lesion at the greater curvature of the lower body. The patient refused further evaluation and follow-up.

### 3.3 Comparison of clinical outcomes after ESD and surgery

**Figure 2:**
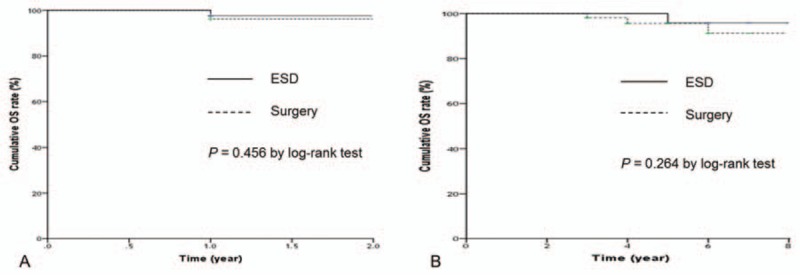
Comparisons of overall survival: (A) less than 2-year follow-up period; (B) more than 2-year follow-up period. ESD = endoscopic submucosal dissection, OS = overall survival.

**Figure 3:**
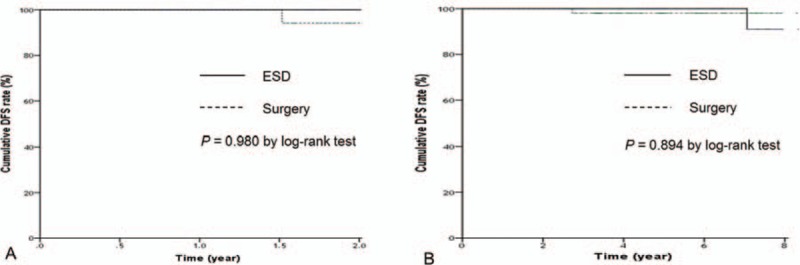
Comparisons of disease-free survival: (A) less than 2-year follow-up period; (B) more than 2-year follow-up period. DFS = disease-free survival, ESD = endoscopic submucosal dissection.

The patients who had follow-up interval less than 2 years were 22 (29.7%) in the ESD group and 9 (11.4%) in the surgery group, respectively. Thus, the OS and the DFS were analyzed according to the follow-up interval. The 2-year OS rate was 97.6% in the ESD group and 92.4% in the surgery group (Fig. 2A), and the 5-year OS rate was 95.8% in the ESD group and 95.6% in the surgery group (Fig. 2B). The 2-year and 5-year DFS rate was 100% in the ESD group. The 2-year DFS was 94.1% and 5-year DFS was 98.4% in the surgery group (Fig. 3). Kaplan–Meier analysis showed no significant difference in 2-year (*P* = .45) and 5-year OS (*P* = .26) or 2-year (*P* = .98) and 5-year DFS (*P* = .89) between the 2 groups. There was no gastric cancer-related mortality during the follow-up period. One patient (1.4%) in the ESD group had a local recurrence (*P* = .46) that met the absolute indication for endoscopic resection after 2 years of primary treatment and was successfully treated with a repeated ESD. Table 2 details the analysis of recurrent and metachronous cancers. Two patients (2.7%) in the ESD group developed metachronous cancer without lymph node metastasis more than 5 years after treatment. Those 2 patients underwent laparoscopic-assisted distal gastrectomy: 1 patient had an undifferentiated tumor histology and the other had an indistinguishable tumor margin due to surrounding mucosal metaplasia. No significant difference was found regarding local recurrence (*P* = .46) and metachronous cancer between the 2 groups (*P* = .22). No other metastasis was observed in either group.

**Table 2:**
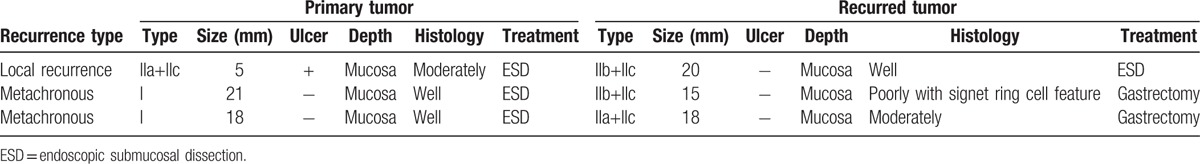
Analysis of recurrent and metachronous cancers in the ESD group.

### 3.4 Comparison of treatment-related adverse events

Treatment-related adverse events are summarized in Table 3. Early adverse events occurred in 9 patients (12.2%) in the ESD group, and in 5 patients (6.3%) in the surgery group. There was no significant difference in the early complication rate (*P* = .21) or Clavien–Dindo grade III or higher complications (*P* = .36) between the 2 groups. The most common early adverse event in the ESD group was bleeding followed by gastric perforation. All patients with bleeding or perforation after ESD were managed successfully with endoscopic intervention and conservative treatment. Two patients in the surgery group required additional surgery under general anesthesia within 3 months of the primary surgery because of wound leakage, and 1 patient developed acute kidney injury due to severe bleeding that required hemodialysis.

**Table 3:**
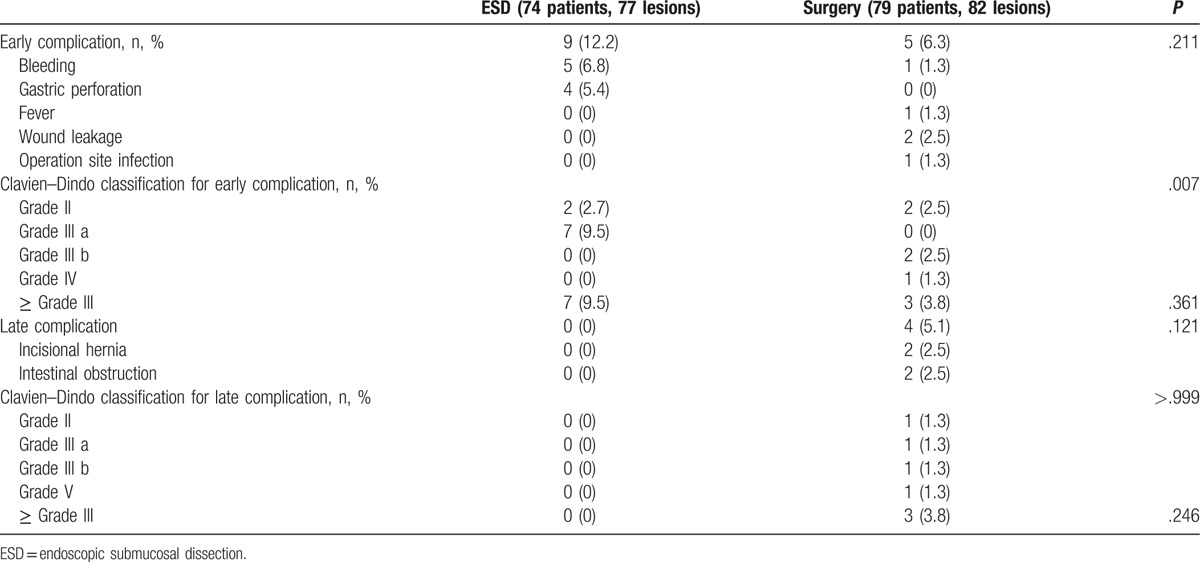
Comparisons of treatment-related adverse events.

Late complications occurred only in the surgery group; however, no significant difference was found regarding the rate of late complications (*P* = .12) and Clavien–Dindo grade III or higher complications (*P* = .24) between the 2 groups. Two patients required additional surgical treatment for incisional hernia repairs, and 1 patient died after an emergency operation for an intestinal obstruction that occurred 3 years after the initial surgery. All surgery-related complications occurred in patients who underwent laparoscopic surgery.

## 4 Discussion

The short-term outcomes of ESD for EGCs are generally acceptable, yielding an en bloc resection rate of 94.9% and a complete resection rate of 94.7%.^[20]^ Our study showed similar results; the en bloc resection rate was 92.2%, and the complete resection rate was 93.5%. Our study also found that long-term clinical outcomes such as local recurrence, metachronous cancer, OS rate, DFS, and late adverse events after ESD were comparable to those of surgery for the treatment of EGC, including the expanded indications. A limited number of studies have reported a direct comparison of long-term outcomes between endoscopic resection and surgery for EGCs. A recent study^[21]^ showed that the 5-year OS rates after endoscopic resection or surgery were 97.5% and 97.0%, respectively, and that there was no significant difference between the 2 groups. The 5-year recurrence rate for gastric cancer, however, was higher in the endoscopic resection group than in the surgery group (4.8% vs 0.3%, respectively); this was likely owing to metachronous cancers in the endoscopic resection group. ESD was not available prior to 2004 and therefore 10.9% of patients in the endoscopic resection group in that study underwent EMR, which may complicate the direct comparison of ESD and surgery. Another study^[22]^ reported that the 3-year survival rates after ESD or surgery were 94.6% and 89.7%, respectively (*P* = .44). That study, however, included both EGCs and dysplastic lesions, and the indications for ESD for EGCs were not disclosed. Many studies including ours discussed the feasibility of expanding indications for EGCs; however, there are several concerns, especially the risk of lymph node metastasis that needs to be addressed. Lymph node metastasis was found in 15.0% of SM cancers that are less than 3 cm and without lymphovascular invasion^[14]^ and 1.7% of lymph node metastases were noted in SM cancers less than 2.0 cm in size^[23]^. Therefore, a combination of ESD and laparoscopic lymph node dissection has been suggested to eliminate the potential risk of lymph node metastasis.^[24]^

Bleeding and micro-perforations are the most common early complications of ESD.^[25,26]^ In our study, bleeding was the most common complication (6.8%), followed by perforation (5.4%). All ESD-related adverse events were managed successfully with conservative or endoscopic treatment, whereas long-term adverse events, which occurred only in the surgery group (5.1%), required additional surgical treatment. Similar to our results, a recent study reported that early complication rates were similar for ESD and surgery (5.5% vs 6.8%, respectively: *P* = .55) with late complications found only in the surgery group (4.8%, *P* = .004).^[21]^ Laparoscopic surgery-related mortality is reported to be 1.1% to 3.3% with a perioperative complication rate of 10.4%.^[27,28]^ The most common complication and cause of death from laparoscopic surgery is anastomotic fistula formation, which occurs in 3.0% to 6.6% of patients during the perioperative period. In our study, among patients in the surgery group who had long-term adverse events, 1 patient (1.3%) died of intestinal obstruction-related sepsis 3 years after the primary surgery. Although there was no significant difference in the complication rate between the patients treated with ESD and surgery, complications associated with ESD had a tendency to be managed with noninvasive methods.

This study has several limitations. First, as the data were analyzed retrospectively, they may have some bias. For example, the ESD group could have positively biased results because the portions of smaller lesions and nonmalignant lesions observed via pretreatment histology were higher in the ESD group. However, considering that tumor size is not included for evaluating tumor stage, it might not have a significant influence on the results. And other than tumor size and pretreatment histology, both groups had similar baseline characteristics, which reduced selection bias and may be an advantage of our study. Second, patients were enrolled at a single institute, and the number of patients enrolled was relatively small owing to limitations placed on the follow-up duration. The follow-up duration differed between patients treated with ESD and surgery, and it is possible that the median follow-up duration was not sufficient to evaluate long-term outcomes. The main cause of follow-up loss is likely the wide availability of endoscopic evaluation in local clinics. In addition, the less-invasive nature of ESD may result in lower compliance with follow-up care. Subgroup analysis for each indication between the 2 groups could not be conducted because of the relatively small number of patients in each group. Lastly, similar to almost all other studies, our study was conducted employing post-treatment pathological specimens. If indications for ESD are defined according to characteristics observed with pretreatment endoscopy, this could be a more reliable study for evaluate whether pretreatment endoscopy-based findings could be used instead of posttreatment histologic specimens.

With the increasing availability of cancer surveillance and the advantages of therapeutic endoscopic treatment, the proportion of EGC treated with ESD is expected to increase. ESD may be a comparable choice, instead of surgery, to treat EGCs that meet the absolute and expanded indications.
